# Systemic lupus erythematosus patients have unique changes in serum metabolic profiles across age associated with cardiometabolic risk

**DOI:** 10.1093/rheumatology/kead646

**Published:** 2023-12-04

**Authors:** Elizabeth C Jury, Junjie Peng, Alexandra Van Vijfeijken, Lucia Martin Gutierrez, Laurel Woodridge, Chris Wincup, Ines Pineda-Torra, Coziana Ciurtin, George A Robinson

**Affiliations:** Centre for Rheumatology Research, Division of Medicine, University College London, London, UK; Centre for Adolescent Rheumatology Versus Arthritis, Division of Medicine, University College London, London, UK; Centre for Rheumatology Research, Division of Medicine, University College London, London, UK; Centre for Rheumatology Research, Division of Medicine, University College London, London, UK; Centre for Adolescent Rheumatology Versus Arthritis, Division of Medicine, University College London, London, UK; Centre for Experimental & Translational Medicine, Division of Medicine, University College London, London, UK; Institute of Structural and Molecular Biology, Division of Biosciences, University College London, London, UK; Centre for Rheumatology Research, Division of Medicine, University College London, London, UK; Centre for Experimental & Translational Medicine, Division of Medicine, University College London, London, UK; Centre for Rheumatology Research, Division of Medicine, University College London, London, UK; Centre for Adolescent Rheumatology Versus Arthritis, Division of Medicine, University College London, London, UK; Centre for Rheumatology Research, Division of Medicine, University College London, London, UK; Centre for Adolescent Rheumatology Versus Arthritis, Division of Medicine, University College London, London, UK

**Keywords:** SLE, age, metabolism, metabolomics, lipids, comorbidities, cardiovascular disease, atherosclerosis, cardiometabolic

## Abstract

**Objectives:**

Cardiovascular disease through accelerated atherosclerosis is a leading cause of mortality for patients with systemic lupus erythematosus (SLE), likely due to increased chronic inflammation and cardiometabolic defects over age. We investigated age-associated changes in metabolomic profiles of SLE patients and healthy controls (HCs).

**Methods:**

Serum NMR metabolomic profiles from female SLE patients (*n* = 164, age = 14–76) and HCs (*n* = 123, age = 13–72) were assessed across age by linear regression and by age group between patients/HCs (Group 1, age ≤ 25, *n* = 62/46; Group 2, age = 26–49, *n* = 50/46; Group 3, age ≥ 50, *n* = 52/31) using multiple *t* tests. The impact of inflammation, disease activity and treatments were assessed, and UK Biobank disease-wide association analysis of metabolites was performed.

**Results:**

Age-specific metabolomic profiles were identified in SLE patients *vs* HCs, including reduced amino acids (Group 1), increased very-low-density lipoproteins (Group 2), and increased low-density lipoproteins (Group 3). Twenty-five metabolites were significantly altered in all SLE age groups, dominated by decreased atheroprotective high-density lipoprotein (HDL) subsets, HDL-bound apolipoprotein (Apo)A1 and increased glycoprotein acetyls (GlycA). Furthermore, ApoA1 and GlycA were differentially associated with disease activity and serological measures, as well as atherosclerosis incidence and myocardial infarction mortality risk through disease-wide association. Separately, glycolysis pathway metabolites (acetone/citrate/creatinine/glycerol/lactate/pyruvate) uniquely increased with age in SLE, significantly influenced by prednisolone (increased pyruvate/lactate) and hydroxychloroquine (decreased citrate/creatinine) treatment and associated with type 1 and type 2 diabetes by disease-wide association.

**Conclusions:**

Increasing HDL (ApoA1) levels through therapeutic/nutritional intervention, whilst maintaining low disease activity, in SLE patients from a young age could improve cardiometabolic disease outcomes. Biomarkers from the glycolytic pathway could indicate adverse metabolic effects of current therapies.

Rheumatology key messages

- Patients with SLE have reduced ApoA1 (HDL) and elevated GlycA levels at all ages.
- ApoA1 and GlycA levels associate with disease activity measures and atherosclerosis and myocardial infarction risk.
- Glycolysis pathway metabolites increase with age in SLE associated with disease treatment and diabetes risk.

## Introduction

Systemic lupus erythematosus (SLE) is an autoimmune disease that affects ∼1 in 1000 people in the United Kingdom who are primarily women [1]. The disease is characterised by autoantibodies targeting nuclear components with a pattern of disease flares. Despite recent improvements in therapeutics, mortality in SLE patients remains high, where cardiovascular disease (CVD) remains the greatest cause and the relative risk of mortality is exacerbated for younger patients with juvenile-onset SLE (JSLE) [2–4].

Atherosclerosis is the leading cause of CVD in the general population and is more prevalent in SLE patients with mechanisms initiating earlier in life compared with healthy individuals [4–6]. Between the ages of 35–44, women with SLE have a 50-fold increased CVD-risk compared with healthy individuals [7]. Separately, JSLE is associated with a 100–300-fold increased CVD mortality risk in young patients compared with age-matched controls [8], and JSLE patients are typically younger if a first CVD event occurs (average 32.2 years) compared with patients with adult-onset SLE (average 48.1 years) [9]. This highlights a need for improved early monitoring of atherosclerosis in SLE [10].

Traditional CVD risk factors fail to explain the elevated risk for SLE patients [11]. Some SLE-specific factors such as corticosteroid use, presence of anti-phospholipid antibodies, and ongoing disease activity/inflammation have been associated with increased CVD risk. In addition, dyslipidaemia, characterised by elevated triglycerides (TGs), and atherogenic very-low-density lipoprotein (VLDL) and low-density lipoprotein (LDL) [both apolipoprotein (Apo)B expressing], as well as decreased atheroprotective ApoA1 expressing high-density lipoprotein (HDL) concentrations are also common in SLE and associated with markers of subclinical atherosclerosis [12]. A ‘lupus pattern’ of dyslipoproteinemia was described in 1997 by Borba and Bonfá of high levels of VLDL cholesterol and TGs and low levels of HDL cholesterol, which was exacerbated by active disease and associated with vasculitis [13]. In support, the Systemic Lupus International Collaborating Clinics (SLICC) cohort analysis of 918 SLE patients showed that 36% had dyslipidaemia at diagnosis, a proportion which raised to over 60% after 3 years [14, 15]. Age of onset, burden of steroid use and lack of antimalarial therapies have been speculated to contribute to this increased prevalence. A study in adult SLE patients showed that traditional CVD risk factors, estimated by the Framingham score which incorporates LDL assessment, were not associated with SLE disease activity [16, 17]. Thus, HDL and ApoA1 could be more relevant players in the mechanisms of premature atherosclerosis due to SLE disease itself.

Using nuclear magnetic resonance (NMR) metabolomics, altered lipid profiles have been shown to occur from a young age in JSLE patients, largely through decreased HDL compared with healthy individuals, as well as increased VLDL in response to a disease flare [18]. In addition, increased circulating ApoB : ApoA1 ratio can predict CVD risk in JSLE patients [19]. In adult patients, decreased medium-HDL measures, as well as increased small-HDL, VLDL and intermediate density lipoprotein (IDL) particles can predict the presence of subclinical atherosclerosis [20]. It is known that age has an impact on metabolism in health and disease which has led to community-based ‘MetaboAge’ predictors for cardiometabolic health using metabolomics [21]. The impact of metabolic profiles on cardiometabolic heath across age in SLE, where persistent chronic inflammation occurs, is yet to be explored.

Here, the metabolome of 164 SLE patients was analysed using NMR spectroscopy to investigate differences in cardiometabolic risk factors between patients with SLE and healthy individuals across age. Age was used as both a categorical and continuous variable. Analysis revealed both age-unique and age-shared metabolite profiles in SLE patients associated with measures of disease activity and CVD risk, as well as metabolites that increased with age in SLE patients only, which were associated with different treatments. These findings could inform future age-tailored therapeutic approaches to reduce CVD in SLE across the disease life-course.

## Methods

### Patients and healthy cohorts

Peripheral blood was collected, and serum was processed from patients attending adult, young adult or adolescent SLE clinics at University College London Hospital (UCLH) fulfilling both the American College of Rheumatology (ACR) classification criteria for SLE (1997) [22] or the SLICC criteria (2012) [23]. Healthy control (HC) blood was collected from volunteers at University College London and during public engagement events. All participants were female. This study had research ethics committee (REC) approval and informed written consent was acquired from both patients and HCs under the ethical approval reference: REC11/LO/0330 or REC15/LO/2065, London-Harrow Research Ethics Committee. All information was stored as pseudo-anonymised data. Clinical data was recorded from patient files and questionnaires (Supplementary Table S1, available at *Rheumatology* online). To minimise pubertal impacts on metabolic profiling, we included only individuals who self-reported completion of puberty (Tanner stage 4–5). Disease activity was calculated using SLE Disease Activity Index (SLEDAI-2K) or British Isles Lupus Assessment Group (BILAG) index; a SLEDAI score ≥6 or global BILAG-2004 score ≥8 (more than one B score in at least one domain) were used to indicate clinically active disease [24]. Patients and HCs were grouped by age for analysis (Table 1). The study design can be found in Supplementary Fig. S1, available at *Rheumatology* online.

**Table 1.** Demographic and clinical characteristics of SLE patients and healthy controls

	Group 1 (≤25 yrs) HC/SLE	Group 2 (26–49 yrs) HC/SLE	Group 3 (≥50 yrs) HC/SLE	*P* value HC *vs* SLE
Total number	46/62	46/50	31/52	—
Age, mean (SD)	20.22 (3.48) / 19.21 (2.85)	36.74 (7.09) / 38.34 (6.31)	62.87 (7.26) / 58.48 (5.33)	Group 1: 0.10^a^ Group 2: 0.24^a^ Group 3: 0.0022^a^
Sex, % female	100	100	100	All groups: >1.00^b^
**Race, number (%)**				
White	26 (57) / 22 (35)	34 (74) / 19 (38)	14 (45) / 27 (52)	Group 1: 0.033^b^ Group 2: 0.00050^b^ Group 3: 0.65^b^
Asian	13 (28) / 19 (31)	3 (7) / 15 (30)	5 (16) / 8 (15)	Group 1: 0.83^b^ Group 2: 0.0037^b^ Group 3: 1.00^b^
Black	3 (7) / 16 (26)	6 (13) / 12 (24)	7 (23) / 14 (27)	Group 1: 0.0092^b^ Group 2: 0.20^b^ Group 3: 0.80^b^
Mixed	4 (9) / 5 (8)	3 (7) / 4 (8)	5 (16) / 3 (6)	Group 1: 1.00^b^ Group 2: 1.00^b^ Group 3: 0.14^b^
**Disease characteristics**	**SLE**	**SLE**	**SLE**	**Between SLE age groups**
Disease duration, years, mean (SD)	6.95 (3.79)	14.24 (7.03)	24.71 (11.18)	<0.0001^c^
SLEDAI, median (IQR)	2 (0–4)	4 (0–6)	0 (0–2)	Group 1 *vs* 3: 0.00020^c^
Global BILAG score, median (IQR)	0 (0–1)	2 (0.75–9)	1 (0–2)	Group 1 *vs* 2: 0.033^c^ Group 2 *vs* 3: 0.010^c^
Active disease (SLEDAI ≥6 or BILAG ≥8), number (%)	14 (23)	23 (46)	7 (13)	0.0007^d^
Lupus nephritis, number (%)	18 (29)	18 (36)	14 (27)	0.58
**Serology, mean (SD)**				
dsDNA (IU/mL) (NR = <50)	441.3 (2248)	254.4 (473.5)	66.50 (123.3)	0.62^c^
ESR (mm/h) (NR=<20)	23.49 (25.71)	17.73 (13.78)	19.11 (16.80)	0.58^c^
C3 (g/L) (NR = 0.9–1.8)	0.98 (0.29)	0.97 (0.29)	1.13 (0.25)	Group 1 *vs* 3: 0.0098^c^ Group 2 *vs* 3: 0.0050^c^
Lymphocyte Count (10^9^/L) (NR = 1.3–3.5)	1.35 (0.63)	1.37 (0.91)	1.53 (0.70)	0.60^c^
**Current SLE treatment, *n* (%)**				
Hydroxychloroquine	55 (89)	33 (66)	33 (63)	0.0031^d^
Prednisolone	31 (50)	38 (76)	37 (71)	0.0082^d^
Mycophenolate mofetil	22 (35)	11 (22)	5 (10)	0.0048^d^
Methotrexate	9 (15)	4 (8)	1 (2)	0.056^d^
Azathioprine	14 (23)	9 (18)	8 (15)	0.61^d^
Rituximab (ever)	20 (32)	16 (32)	22 (42)	0.45^d^
**Cardiovascular risk factors, *n* (%)**				
Dyslipidaemia	19 (31)	27 (54)	33 (63)	0.0014^d^
All previous cardiovascular events	0 (0)	5 (10)	4 (8)	0.049^d^
Previous stroke	0 (0)	5 (10)	1 (2)	0.014^d^
Previous myocardial infarction	0 (0)	0 0	3 (6)	0.037^d^
Hypertension	10 (16)	6 (12)	14 (27)	0.13^d^
Antiphospholipid syndrome (positive test)	1 (2)	1 (2)	3 (6)	0.38^d^
Current statin treatment	0 (0)	1 (2)	6 (12)	0.0063^d^
Smoking current	0 (0)	3 (6)	6 (12)	0.0260^d^
Smoking ever	0 (0)	2 (4)	10 (19)	0.00020^d^
BMI, mean (SD)	24.12 (5.34)	25.09 (5.52)	25.09 (4.10)	0.80^c^
**Diabetes disease characteristics**				
All diabetes	1 (2)	0 (0)	4 (8)	0.055^d^
Type I diabetes	0 (0)	0 (0)	1 (2)	0.34^d^
Type II diabetes	1 (2)	0 (0)	3 (6)	0.15^d^
Insulin	0 (0)	0 (0)	1 (2)	0.34^d^
Metformin	0 (0)	0 (0)	1 (2)	0.34^d^

a *t* test.

b Fishers exact test.

c One-way ANOVA.

d χ^2^ test.

C3: Complement component 3; dsDNA: anti-double-stranded-DNA antibodies; ESR, erythrocyte sedimentation rate; NR: normal ranges; SLEDAI: Systemic Lupus Erythematosus Disease Activity Index.

### Metabolomics

Serum metabolomic analysis was performed using NMR spectroscopy by Nightingale Health (https://nightingalehealth.com/), enabling the simultaneous measurement of amino acids, fatty acids, glycolysis metabolites, total lipid measures and in-depth lipoprotein measurements including particle size and content (Supplementary Table S2, available at *Rheumatology* online). Disease-wide association analysis of metabolites was performed using the Nightingale Atlas webtool which scans matched metabolites quantified for ∼120 000 participants in the UK Biobank against prevalence, incidence and mortality of over 700 common diseases (endpoints derived from UK Hospital Episode Statistics data and national death registries) [25]. Metaboanalyst (https://www.metaboanalyst.ca/home.xhtml) was used to assess metabolic pathways and networks.

### Statistical analysis

Statistical analysis was performed using GraphPad Prism 10. Data was tested for normal distribution and parametric/non-parametric tests were used accordingly. Unpaired two-tailed *t* tests, Mann–Whitney test, and one-way ANOVA (Turkey’s post-hoc test) were used as appropriate. Multiple testing was accounted for using the false discovery rate (FDR) adjustment for multiple comparisons (Benjamini, Krieger and Yekutieli approach) of *P* values (1%). χ^2^d, Fishers exact test, or unpaired *t* tests were appropriately employed for analysing demographic features. Receiver operating characteristic (ROC) analysis was used for diagnostic assessment of sensitivity and specificity for markers of interest. Pearson’s correlations were computed with two-tailed *P* values calculated with a 95% CI. Chord plots were produced using the ‘circlize’ package in R to summarise correlations (Pearson correlation coefficients) between selected metabolites and clinical factors [26].

## Results

### Patients with SLE have unique changes in serum metabolites by age group compared with healthy individuals

NMR metabolomics was performed on 164 female patients with SLE and 123 matched HCs. Patients and HCs were split by age into three groups based on sample distribution and physiological/social age brackets: Group 1 (≤25 years, *n* = 62 and 43), Group 2 (26–49 years, *n* = 50 and 46) and Group 3 (≥50 years, *n* = 52 and 31) (Table 1; Supplementary Table S1, available at *Rheumatology* online). Despite the low median disease activity scores for the full SLE cohort (2 for SLEDAI and 1 for global BILAG), patients in Group 2 had a higher median disease activity and proportion of patients with active disease compared with other groups, whilst Group 1 and 2 had significantly lower C3 compared with Group 3. Group 1 had a significantly higher proportion of patients treated with hydroxychloroquine compared with Group 2 and 3, which had a higher proportion of patients on prednisolone. The proportion of patients on mycophenolate mofetil reduced progressively from SLE age Group 1 through to Group 3. The proportion of patients with dyslipidaemia determined as per routine test cut-offs (48% of the SLE cohort), on statins (4% of the SLE cohort), and with a smoking history (7% of the SLE cohort) significantly increased from Group 1–3. There was also a higher percentage of SLE patients with previous CVD events (stroke/myocardial infarction) observed for Group 2 (10%) and Group 3 (8%) compared with Group 1 (0%), and most patients with diabetes were in Group 3 (8%).

**Figure 1.**
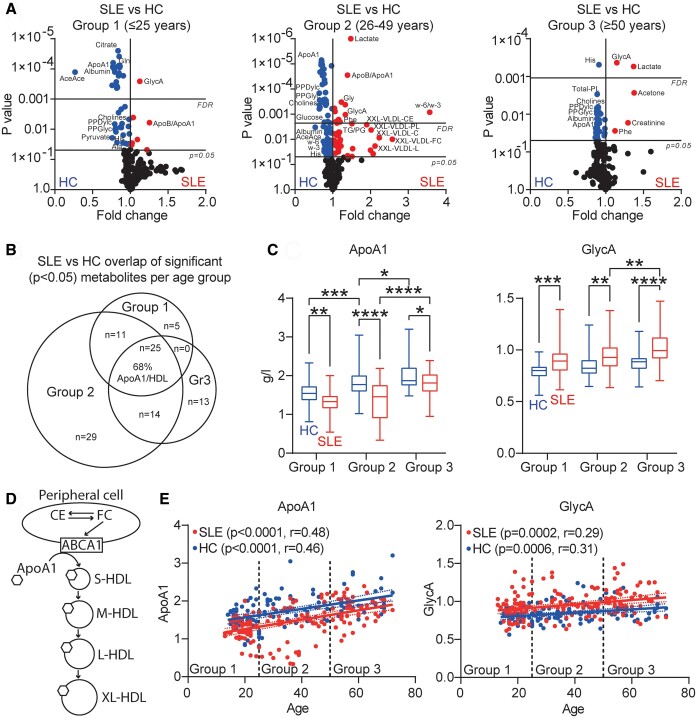
Patients with SLE have unique cardiometabolic changes by age group, but a shared decrease in ApoA1 (HDL) and increase in GlycA across age compared with HCs. **A** Volcano plot showing the fold change of serum metabolites between SLE patients and HCs in different age groups: Group 1 (≤25 years, *n* = 62 and 46), Group 2 (26–49 years, *n* = 50 and 46) and Group 3 (≥50 years, *n* = 52 and 31). Log10 *P* values are displayed. Horizontal line represents adjusted *P* value threshold following 1% false discovery rate adjustment for multiple comparisons. **B** Venn diagram displaying the proportional overlap of statistically significantly altered (*P* < 0.05) metabolites from (**A**) that overlap between the different age group comparisons. The percentage of total overlapping metabolites (*n* = 25) that were ApoA1/HDL-associated was 68%. **C** Box and whisker plots displaying concentration of serum ApoA1 and GlycA compared between SLE patients and HCs in each age bracket. Unpaired *t* test. Mean. SEM. *P < 0.05, **P < 0.01, ***P < 0.001, ****P < 0.0001. **D** Schematic representation of peripheral cell lipid efflux to apolipoprotein (Apo)A1 to produce circulating high density lipoproteins (HDL). **E** Pearson correlations between ApoA1 or GlycA and age in SLE patients (*n* = 164) and HCs (*n* = 123). *P* and r values are displayed. ApoA1: apolipoprotein A1; ABCA1: ATP Binding Cassette Transporter A1; CE: cholesterol esters; FC: free cholesterol; HDL: high density lipoproteins; L: large; M: medium; S: small; VLDL: very low density lipoproteins; XL: very large

Metabolomics data was analysed by FDR-corrected multiple *t* tests, comparing SLE patients to HCs in each age bracket (Fig. 1A, Supplementary Table S3, available at *Rheumatology* online). In Group 1, glycoprotein acetyls (GlycA) was the most significantly increased metabolite in SLE patients compared with HCs, whilst a greater number of metabolites were decreased in concentration, including citrate, glutamine, albumin, acetoacetate, ApoA1 and multiple HDL particle measures. For Group 2, the most significantly increased metabolites were lactate and the ApoB : ApoA1 ratio, followed by glycine, GlycA, and the omega-6:3 ratio. Again, a greater proportion of metabolites were decreased in SLE patients compared with HCs, including ApoA1, multiple HDL metabolites, phosphatidylcholine, phosphoglyceride, cholines and glucose. Finally, in Group 3, GlycA, lactate, acetone, creatinine and phenylalanine were the most upregulated in SLE, whilst histidine, choline, phosphatidylcholine, phosphoglyceride, albumin, ApoA1 and multiple HDL metabolites were downregulated.

Metabolites significantly altered in SLE patients unique to each age group were classified using Venn analysis (Fig. 1A and B; Supplementary Table S4, available at *Rheumatology* online). Group 1 had only five differentially expressed metabolites in SLE compared with age-matched HCs, including reduced alanine, glutamine and pyruvate, and increased LDL size, whereas Group 2 had the highest number of differentially expressed metabolites (*n* = 29) including glycine, acetate, omega-6:3 ratio, total fatty acids (FAs), polyunsaturated FAs (PUFAs), the TG:(phosphoglyceride)PG ratio and multiple extremely large VLDL metabolites were increased, whilst DHA, glucose and multiple very large HDL metabolites were decreased. Finally, in Group 3, acetone and creatinine were increased, whilst multiple total and large/medium/small-sized LDL metabolites were decreased. Some serum metabolites were shared exclusively across two age groups, such as increased lactate and phenylalanine in SLE patients in both Group 2 and 3, compared with reduced acetoacetate and increased ApoB : ApoA1 ratio in both Group 1 and 2, supporting a more conserved signature across consecutive age groups in SLE for some serum metabolites.

Together, this supports a need for age consideration in SLE biomarker research.

### Patients with SLE have reduced HDL and ApoA1 and increased GlycA across all age groups

In addition to age-unique metabolites altered in SLE compared with HCs, a subset of 25 metabolites were commonly dysregulated in SLE across all three age groups (Fig. 1B; Supplementary Table S4, available at *Rheumatology* online). Twenty-four of these metabolites were significantly reduced in SLE compared with HCs at all ages, dominated by total, small and medium HDL metabolites and HDL-bound peptide ApoA1, representing 68% of the 25 age-common metabolites (Fig. 1B and C; Supplementary Table S4, available at *Rheumatology* online). Using ApoA1 as a global marker for total HDL expression, the greatest reduction in SLE was in Group 2 (*P* < 0.0001), followed by Group 1 (0.00020), and then Group 3 (*P* = 0.018) (Fig. 1C), which was supported by ROC analysis (AUC = 73.70, 71.40 and 60.76, respectively, and AUC = 67.38 for all patients combined when compared with HCs, Supplementary Fig. S2A, available at *Rheumatology* online). The mechanism of peripheral cell lipid efflux to ApoA1 to produce circulating HDL of different sizes is shown in Fig. 1D. Other metabolites commonly decreased in SLE patients across age included albumin, histidine, choline, phosphatidylcholine, phosphoglyceride, as well as total particle concentration and phospholipid content of lipoproteins. Strikingly, the only increased metabolite shared across all age groups in SLE was GlycA (Group 1, *P* = 0.00030; Grpup-2, *P* = 0.0023; Group 3, *P* = 0.00040. Fig. 1B and C) highlighting a possible indicator of inflammation across age. For GlycA, Group 3 had the highest AUC (74.01) by ROC analysis compared with the other SLE age groups and all SLE patients combined when compared with HCs (Supplementary Fig. S2A, available at *Rheumatology* online). Interestingly, whilst ApoA1 and GlycA did not correlate significantly with each other (Supplementary Fig. S2B, available at *Rheumatology* online), both correlated positively with age in both HCs and SLE patients and there was no significant difference in linear regression slopes between SLE and HCs, suggesting the same rate of increase with age (Fig. 1E); however, the distances between linear regression curves for ApoA1 and GlycA were consistently lower and higher, respectively, for SLE patients compared with HCs across age, supporting their consistent dysregulation in patients.

### HDL, ApoA1 and GlycA are differentially associated with measures of disease activity in SLE across age and risk of cardiovascular comorbidities

**Figure 2.**
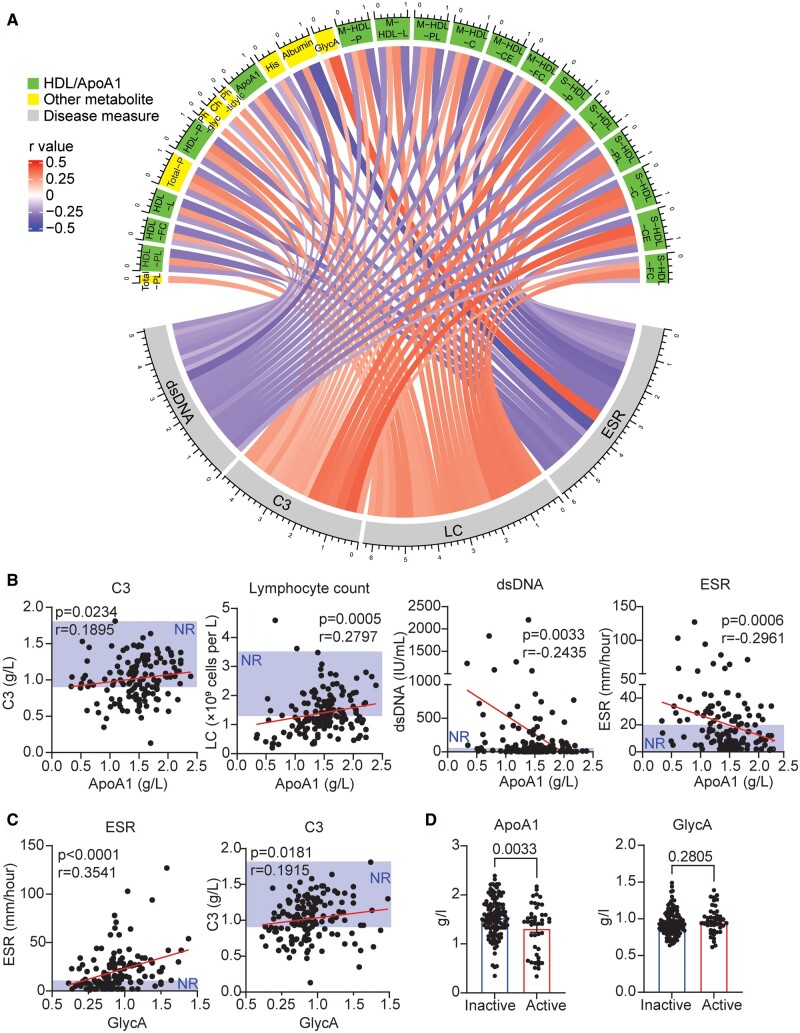
ApoA1 and GlycA are differentially associated with measures of SLE disease activity. **A** Circos plot displaying correlations between the 25-metabolite signature associated with SLE in all age groups and clinical serological measures of SLE disease. Pearson correlation coefficients are represented as edges connecting the metabolites and clinical factors. The width of the edges represents the size of the correlation. Only statistically significant correlations following adjustment for multiple comparisons (10% FDR) are shown. Red line represents positive correlation and blue line represents negative correlation. **B**–**C** Pearson correlations between serum (**A**) ApoA1 or (**B**) GlycA and serological measures of SLE disease in SLE patients (*n* = 164). *P* and r values are displayed. NR: normal range. **D** Scatter plots with histograms displaying concentration of serum ApoA1 and GlycA compared between SLE patients with active (*n* = 44) and inactive (*n* = 120) disease, with active defined by SLEDAI ≥6 or BILAG ≥8. Unpaired *t* test. Mean. SEM

Patients were combined (*n* = 164) and the 25 overlapping metabolites commonly dysregulated across all ages in SLE were correlated with serological measures of disease activity (Fig. 2A). HDL metabolites and ApoA1 correlated negatively with both dsDNA and ESR and positively with both lymphocyte count and C3 levels (Fig. 2A and B), whereas GlycA correlated positively with erythrocyte sedimentation rate (ESR, a non-specific measure of inflammation) and C3 (Fig. 2A and C), suggesting that ApoA1 (HDL) is more significantly associated with SLE-specific biomarkers than GlycA. In support, patients with active disease scores had significantly lower ApoA1 levels but no difference in GlycA (Fig. 2D). In contrast, whilst the small proportion of patients on statin therapy had increased ApoA1 levels, ApoA1 was not affected by SLE-specific treatment, whereas GlycA was significantly increased in patients on prednisone and decreased in patients on hydroxychloroquine (Supplementary Fig. S3, available at *Rheumatology* online). Of note, neither metabolite had a significant association with the presence of lupus nephritis (Supplementary Fig. S4A, available at *Rheumatology* online). Thus, ApoA1 (HDL) and GlycA are key metabolites dysregulated across age in SLE patients differentially associated with serological inflammatory measures and treatment. Interestingly, whilst there was no influence of hypertension and smoking status, patients with clinically defined dyslipidaemia had significantly increased GlycA, but no difference in ApoA1, supporting the use of these biomarkers for CVD risk assessment (Supplementary Fig. S4B–D, available at *Rheumatology* online). This was also the case for BMI, where only GlycA had a significant correlation (Supplementary Fig. S4E, available at *Rheumatology* online), despite there being no difference in BMI between the groups (Table 1).

**Figure 3.**
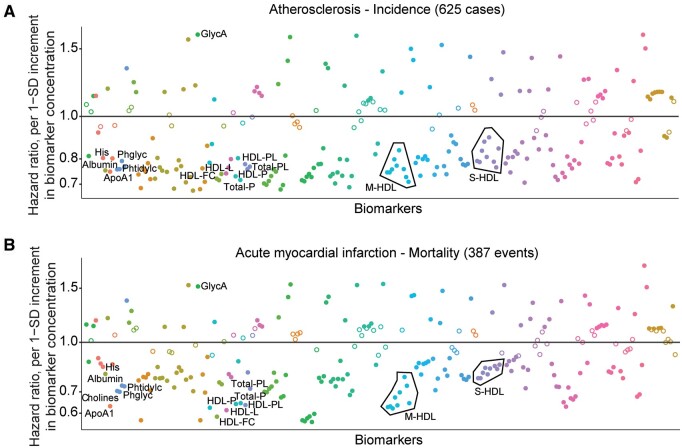
Metabolomic profiles associated with SLE across age are related to cardiovascular disease risk in the UK Biobank. Disease-wide association hazard ratio plots displaying the relationship between the 25-metabolite signature associated with SLE in all age groups and (**A**) atherosclerosis incidence, or (**B**) acute myocardial infarction mortality in the general population by Nightingale Atlas software, which uses open access to results from a disease-wide association scan of blood biomarkers quantified by Nightingale Health for >120 000 participants in the UK Biobank. Licensed under a Creative Commons Attribution Non-Commercial No-Derivatives 4.0 International License (CCBY-NC-ND). Hazard ratio per 1-SD increment in concentration

The 25-metabolite signature was also analysed by Nightingale Atlas UK Biobank disease-wide association analysis [25] (Fig. 3). Strikingly, in the UK Biobank cohort, all 24 metabolites from the signature that were reduced in SLE in all age groups, including all HDL metabolites and ApoA1, had a significant negative association (hazard ratio <1) with both atherosclerosis incidence (from 625 cases) and myocardial infarction mortality (from 387 events), whilst GlycA (increased in SLE) had a significant positive association with both (Fig. 3A and B; Supplementary Table S5, Supplementary Fig. S5A and B, both available at *Rheumatology* online). As a validation, all 25 metabolites were significantly associated with SLE incidence using this analysis from 92 cases (Supplementary Fig. S5C, available at *Rheumatology* online). Thus, early intervention to increase ApoA1 (HDL) and/or reduce GlycA in SLE patients could be key to improving cardiovascular outcomes.

### Metabolites associated with the glycolysis pathway increase with age and are associated with treatment in SLE patients and diabetes risk

**Figure 4.**
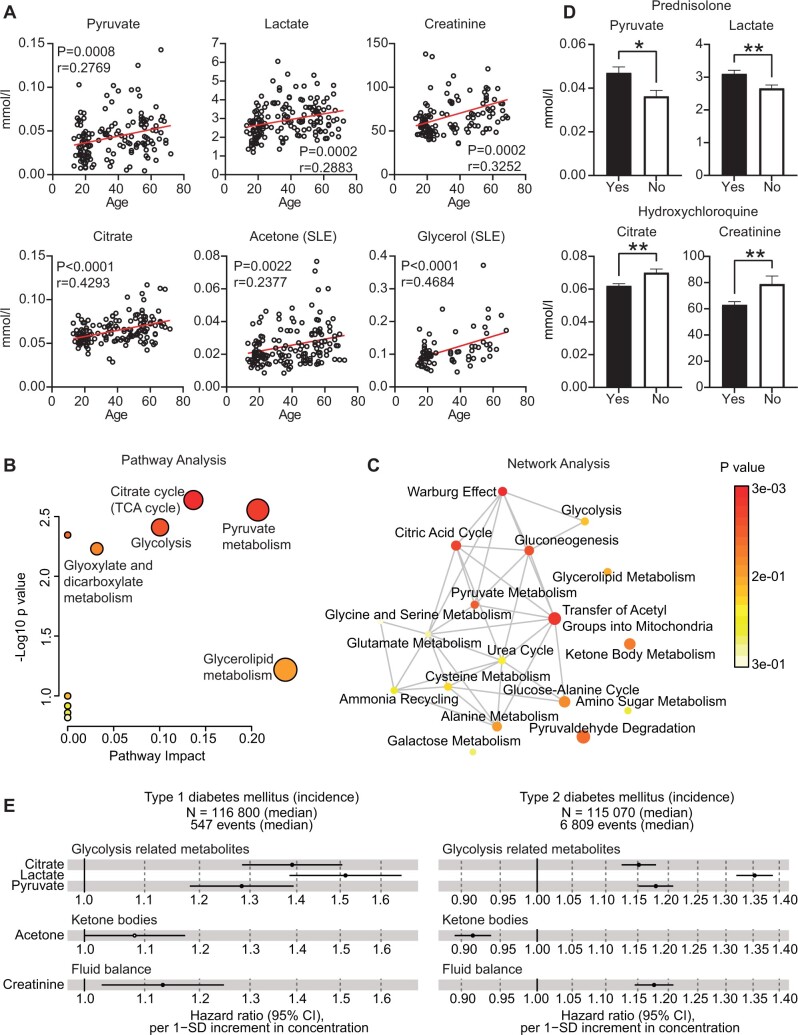
Metabolites that correlate with age in SLE, but not HCs, are associated with glycolysis, treatments and diabetes risk. **A** Pearson’s correlations between serum concentrations of the six SLE-unique metabolites with age in the full cohort of SLE patients (*n* = 164). *P* and r values are displayed. **B** Pathway analysis plot (Metaboanalyst) displaying the pathway impact against -log10 *P*-value of the pathways identified from the six metabolites that correlate with age in SLE only. **C** Network analysis of (**B**), displaying which pathways are metabolically connected with each other. For (**B**) and (**C**), node size and colour reflect the pathway impact and *P*-value, respectively. **D** Histograms displaying the concentration of serum glycolysis metabolites from (**A**) that were significantly impacted when patients were stratified by treatment with either prednisolone (*n* = 106) or hydroxychloroquine (*n* = 121). *P < 0.05, **P < 0.01. **E** Disease-wide association hazard ratio Cox regression plots displaying the relationship between the SLE age/glycolysis-associated metabolites and type I and II diabetes incidence in the general population by Nightingale Atlas software, which uses open access to results from a disease-wide association scan of blood biomarkers quantified by Nightingale Health for >120 000 participants in the UK Biobank. Licensed under a Creative Commons Attribution Non-Commercial No-Derivatives 4.0 International License (CCBY-NC-ND). Hazard ratio per 1-SD increment in concentration. 95% CIs

To extend the age group analysis, correlations of metabolite concentrations with age, treated as a continuous variable, were explored in both HCs and SLE patients. Following FDR correction for multiple testing, 115 metabolites significantly correlated with age in SLE, where six of these correlations were not observed in HCs (Supplementary Fig. 6A(left), available at *Rheumatology* online), suggesting a unique age association with disease. Strikingly, all six metabolites were part of the glycolysis pathway, including acetone, citrate, creatinine, glycerol, lactate and pyruvate (Fig. 4A, Supplementary Fig. 6A(right), available at *Rheumatology* online). This was validated by pathway analysis (Fig. 4B), which highlighted the citrate cycle (*P* = 0.0023), pyruvate metabolism (*P* = 0.0028) and glycolysis (*P* = 0.0039) as the most significantly upregulated pathways. Network analysis also highlighted that these pathways are closely linked with both each other and with a larger metabolic network involving pathways including amino acid metabolism (Fig. 4C).

Finally, despite the six age-associated and SLE-unique metabolites showing no correlation with SLE disease activity measures, pyruvate (*P* = 0.01) and lactate (*P* = 0.009) were significantly increased in patients treated with prednisolone, whilst citrate (*P* = 0.002) and creatinine (*P* = 0.005) were significantly decreased in patients treated with hydroxychloroquine (Fig. 4D). The proportion of younger patients in the study treated with hydroxychloroquine and prednisolone was respectively higher and lower than in the older patient age groups (Table 1), which could explain the age-associated increase in these metabolites and supports the glycolytic impact of these therapies. In addition, disease-wide association analysis showed that these SLE treatment-associated metabolites had a significant positive association with the incidence of both type 1 and type 2 diabetes in the UK Biobank cohort (Fig. 4E), but not with SLE incidence (Supplementary Fig. 6B, available at *Rheumatology* online), suggesting that these treatments may modify the metabolic risk of these comorbidities for patients. In addition, Group 3 had a higher proportion of patients with diabetes (8%), supporting the potential association between the prolonged use of steroid treatment and diabetes risk in SLE, as well as other contributing factors which increase with age (such as increased BMI).

Together, the results show that metabolites of the glycolysis pathway are uniquely associated with ageing in SLE and are influenced by both prednisolone and hydroxychloroquine treatment.

## Discussion

This study provides a novel analysis of the serum metabolome throughout age in SLE patients compared with an age-matched healthy population and is the largest NMR metabolomic cohort study to date in SLE. We identified a common reduction in ApoA1 (HDL) and elevated GlycA levels over age in SLE associated with serological measures of disease activity and cardiovascular disease risk. We also found glycolysis metabolites uniquely correlated with age in SLE and were associated with SLE treatments and diabetes risk in the general population. This highlights a need to monitor and address the cardiometabolic risk of SLE patients from a young age and tailor therapies appropriately.

HDL, typically described as atheroprotective, has previously been shown to be reduced in young SLE patients [16, 17, 27, 28]; of note, we previously applied a similar NMR metabolomic platform analysis to a cohort of JSLE patients, where we identified lower concentrations of circulating small-sized HDL particles compared with HCs, which was exacerbated in patients with high disease activity, described both serologically and by clinical flares [18]. In our current study across age in SLE, we found that both small- and medium-sized HDL subsets, as well as the HDL peptide region, ApoA1, were decreased in SLE patients in all age groups compared with HCs, particularly in the younger age groups and those with active disease, supporting previous studies in JSLE and the ‘lupus pattern’ of dyslipoproteinemia described by Borba and Bonfá [13]. Coupled with chronic inflammation, this could have significant impact on the progression of atherosclerosis throughout life in these patients. Specifically, smaller HDL particles have been shown to have more efficient cholesterol efflux capacity from the liver in mice [29], whilst large- and medium-sized HDL particles have been shown to infer a greater protection from myocardial infarction in humans [30]. In addition, apolipoprotein concentrations and ratios have been shown to reflect subclinical atherosclerotic risk in children [31] and cardiometabolic risk in adults [32] in the general population, as well as in a subset of young JSLE patients [19]. This was supported by disease-wise association analysis in our study using data from the UK Biobank, which showed a strong and significant association between the ApoA1 and HDL-subset signature (reduced levels) and increased risk of atherosclerosis and mortality from myocardial infarction. The SLE-specific mechanisms associated with chronic inflammation could also contribute to dysregulated lipid signatures, where previous studies have suggested that HDL may be reduced in SLE through inflammatory damage of hepatic cells, as suggested by HDL correlation with liver damage serology [18], elevated levels of autoantibodies against ApoA1 [33], impaired cellular efflux of cholesterol to ApoA1 (HDL) [34], and HDL antioxidant capacity [35]. Together, this inflammation induced reduction in HDL levels and the capacity of HDL to carry out its primary atheroprotective functions of reverse cholesterol transport (removal of cholesterol from peripheral tissue) and inhibition of LDL/VLDL (ApoB expressing lipoproteins) oxidation (reducing LDL/VLDL recognition by scavenger receptors), could dramatically increase CVD risk in SLE patients through the increased LDL cholesterol uptake (and reduced lipid efflux) by macrophages in atherosclerotic plaques, coupled with the persistent chronic inflammatory environment of SLE. Here we suggest that these mechanisms accelerate from a young age in SLE.

With respect to ApoB expressing lipoproteins, we found that the ApoB : A1 ratio was increased in the younger Group 1 and Group 2, but not in Group 3. In support of this finding, we have shown previously that JSLE patients with a high ApoB : A1 ratio have an increased cardiometabolic risk and inflammatory profile [19, 36]. Importantly, the Cardiovascular Risk in Young Finns Study showed that an increased ApoB : A1 in healthy young individuals reflected a predisposition to subclinical atherosclerosis in adulthood [37]. Other studies have also supported a role for ApoB : A1 as a biomarker with improved CVD predictive value compared with conventional cholesterol measures [38, 39]. This could be due to the exclusion of VLDL specificity in these measures, which we have previously shown to be increased in adults [20, 40] and young SLE patients [18] with a high disease activity, and may explain the exclusive increase of VLDL subsets in Group 2, who had a significantly greater disease activity compared with the other age groups. This also suggests that the increased ApoB may be driven by VLDL, more than LDL in younger patients, where reduced HDL in circulation could impact the oxidation and atherogenic characteristics of VLDL. Accumulation of TG-rich VLDL particles in SLE may be caused by reduced VLDL catabolism by impaired lipoprotein lipase (LPL) activity, as shown previously in SLE by *in vitro* lipolysis assays and shown to be induced by TNF, IL-1 and IFN-gamma, cytokines that are characteristically high in SLE, leading to both high VLDL-TG and low HDL levels [40, 41]. In addition, ApoA1 has been shown to decrease TNF production via inhibition of contact-mediated activation of monocytes by T cells [42]; thus, low HDL levels in SLE may also exacerbate VLDL accumulation through reduced LPL activity. Although VLDL has been less well studied, due to the focus of clinical measures on LDL and HDL, VLDL particles have also been associated with residual CVD risk and it is speculated that larger VLDL particles may struggle to leave the subendothelial space of arteries, promoting atherosclerotic plaque progression [43, 44]. Alternatively, since ApoA1 in our study was decreased across all ages in SLE and was exacerbated further by active disease, it is also possible that an increased ApoB : A1 ratio could be driven by a dominant decrease in ApoA1, supporting previous work describing reduced HDL to be the most common lipid abnormality in SLE (80% of patients with active disease and approximately one-third of inactive patients) [13].

Regarding the most significant SLE manifestation, we found no relationship between renal involvement history and ApoA1 levels. This was a surprise as previous studies have shown that patients with lupus nephritis have more severe dyslipidaemia and lower HDL levels compared with patients without renal manifestation [45]. Despite finding no significant association between current SLE treatment and HDL levels, it is possible that the altered HDL profile across the cohort could partly be due to the high proportion of patients treated with hydroxychloroquine, as per consensus guidelines, which is known to affect HDL cholesterol levels [46–48]. Specifically, a recent meta-analysis assessing the effect of hydroxychloroquine on lipoprotein levels showed that hydroxychloroquine significantly reduced LDL-C (weighted mean difference, WMD: –0.21 mmol/l, *P* = 0.006), and increased HDL-C concentrations (WMD: 0.03 mmol/l, *P* = 0.03) [46]. Whilst the anti-inflammatory effects of hydroxychloroquine could play a role in this increase in HDL [47], metabolic mechanisms have also been investigated, with a recent study showing that hydroxychloroquine therapy increased the transfer of unesterified cholesterol to HDL *in vitro* [49]. Together, this demonstrates favourable evidence for the use of antimalarials in the treatment of both SLE and atherosclerotic comorbidities. Regarding specific lipid-targeted therapeutics, statins have shown mixed outcomes in both adult SLE and JSLE trials [50–55], and evidence suggests that both statins and PCSK9 therapies have a low impact on HDL particle concentration and cholesterol content [56], providing evidence that new therapeutic strategies to increase HDL for adequate management of atherosclerosis risk are needed both in SLE and in the general population. Patients treated with statins in our cohort had increased ApoA1; however, this analysis was underpowered and could have been an artefact of increased statin use in the older patient group where ApoA1 was naturally higher, matching the increase in clinical dyslipidaemia with age observed in our cohort. Fortunately, therapies that use an intravenous formulation of ApoA1 are showing promise in clinical trails through replacing defective HDL and/or increasing cholesterol efflux capacity to HDL [57].

Strikingly, GlycA was the only metabolite significantly increased in SLE in all age groups compared with HCs, highlighting a possible therapeutic target, which was associated with risk of atherosclerosis and mortality from myocardial infarction in the disease-wise association analysis. Unlike ApoA1 (HDL), GlycA was not affected by disease activity and displayed weak correlations with SLE serological markers. This was surprising, yet important, as GlycA has been shown to be a metabolic biomarker of both chronic inflammation and risk of severe infection [58]. In addition, GlycA has recently been identified as a novel inflammatory biomarker of early CVD risk in young individuals from an adolescent and young adult cohort of 3306 individuals combining the Avon Longitudinal Study of Parents and Children (UK, mean age 15.4 ± 0.3) and the Cardiovascular Risk in Young Finns Study (Finland, mean age 32.1 ± 5.0) [59]. Here, baseline GlycA levels were associated with increased vascular dysfunction (measured by flow‐mediated dilation), and lifestyle‐related CVD and cardiometabolic risk factors over 9 to 10 years of follow‐up and predicted future risk of hypertension and metabolic syndrome. The study also showed that GlycA was a more sensitive measure of early CVD risk stratification than C-reactive protein (CRP) for detecting and stratifying early cardiovascular risk [60], a finding that has been supported by several large cohort studies [61]. Considering that CRP is not usually influenced by SLE-related inflammation, GlycA could be a promising biomarker to stratify patients by for CVD risk. Finally, this multi-cohort study was performed in young, healthy individuals free from established inflammatory disease [59], and GlycA correlated positively with age even in the HCs in our study. Thus, the CVD risk implications of persistently high GlycA levels from a younger age in SLE patients, which increases throughout age, could be significantly greater for long-term outcomes in this patient population. Promisingly, whilst GlycA was significantly increased in patients with dyslipidaemia and correlated positively with BMI, this indicates that lipid lowering therapy and/or dietary intervention to increase weight loss could improve GlycA levels. Finally, as well as reducing the use of steroids to control GlycA levels, our results show that there could also be a key role for the use of antimalarials to play in reducing the levels of GlycA in SLE patients to reduce CVD risk, where levels were reduced in patients treated with hydroxychloroquine. It will be important to understand the mechanisms associated with this beneficial metabolic effect of hydroxychloroquine.

In our study, only six metabolites correlated positively with age in SLE patients, but not HCs, which were exclusive to the upregulated glycolysis pathway, had a significant positive association with diabetes in the general population UK Biobank cohort, and were associated with prednisolone and hydroxychloroquine treatment. Hydroxychloroquine has been shown to have beneficial effects on both lipoprotein and glucose metabolism in autoimmunity [62], whilst prednisolone has been shown to promote fatty acid synthesis and inhibit fatty acid β-oxidation [63]. In addition, corticosteroids can induce higher blood glucose levels, which could lead to steroid-induced type 2 diabetes with long-term use, while hydroxychloroquine has protective effects. Our data suggests that these protective effects of hydroxychloroquine may be induced through the reduction of citrate and creatinine in SLE patients. Despite this, prolonged exposure to these treatments, particularly prednisolone, may explain why these glycolytic metabolites were increased in the two older SLE age groups when compared with HCs by *t* test, but not in Group 1 (≤25 years). Therefore, these glycolytic metabolites could act as biomarkers of adverse treatment effects in patients to prevent comorbidities and improve quality of life.

The sample sizes of previous metabolomics studies in SLE have been generally small, used different platforms, and have failed to take changes with age into account, together resulting in contradictory findings. Addressing these problems are key strengths to our study, as well as our use of NMR spectroscopy, which allows for rapid analysis with high reproducibility of results, whilst requiring little sample preparation and lower costs compared with mass spectrometry [20, 64]. This highlights the translational potential of these identified biomarkers; however, our study also has several limitations to acknowledge. Our study was carried out exclusively in women as our SLE cohort was underpowered for an analysis by sex, due to the profound female predominance of SLE. Despite ensuring all patients were post-pubertal, we did not have information on the menopausal status of age-appropriate women included in this analysis. These are important considerations as we have previously shown that HDL particles are increased by both physiological and therapeutic oestradiol [65], and another study showed that HDL subsets are increased by oestradiol therapy prescribed for menopausal symptoms [66], despite menopause itself having no significant effect on HDL or GlycA. We also observed statistically significant differences in ethnicity, disease activity measures, and treatments between the SLE age groups, which also may have influenced the metabolomic profiles by age, but our study was underpowered for these additional analyses. Despite this, analysis by disease activity, serological measures, and treatment was carried out to enable the data to be interpreted in a clinically relevant way. Finally, this study will benefit from external validation to address geographic, genetic, demographic and socioeconomic influences on metabolism by age in SLE patients for clinical implementation of cardiometabolic risk monitoring in a global setting.

Together, this study highlights key changes in cardiometabolic profiles across age in SLE, concluding that increasing HDL/ApoA1 levels, whilst maintaining low disease activity and minimizing exposure to steroids in SLE patients from a young age could improve cardiometabolic outcomes and mortality. There is an important role to be played for the tailored use of lipid lowering therapies and SLE-targeted antimalarials in controlling HDL, GlycA, citrate and creatinine levels and subsequent CVD and diabetes risk. Finally, metabolic biomarkers from the glycolytic pathway could be used to indicate adverse metabolic effects of current therapies.

## Supplementary material

Supplementary material is available at *Rheumatology* online.

## Supplementary Material

kead646_Supplementary_Data

## Data Availability

Metabolomic data on all patients and controls can be found at Mendeley Data (http://dx.doi.org/10.17632/pngxy9sg9c.1).
