# Supplementary material for: Systemic lupus erythematosus patients have unique changes in serum metabolic profiles across age associated with cardiometabolic risk
Source: Rheumatology (Oxford). 2023 Dec 4;63(10):2741–53. doi: 10.1093/rheumatology/kead646 (PMC11443078; doi:10.1093/rheumatology/kead646)
Supplement: kead646_Supplementary_Data [file kead646_supplementary_data.zip › kead646_Supplementary_Data/rhe-23-1755-File007.docx]

**Supplementary Figures**

**Systemic lupus erythematosus patients have unique changes in serum metabolic profiles across age associated with cardiometabolic risk**

**
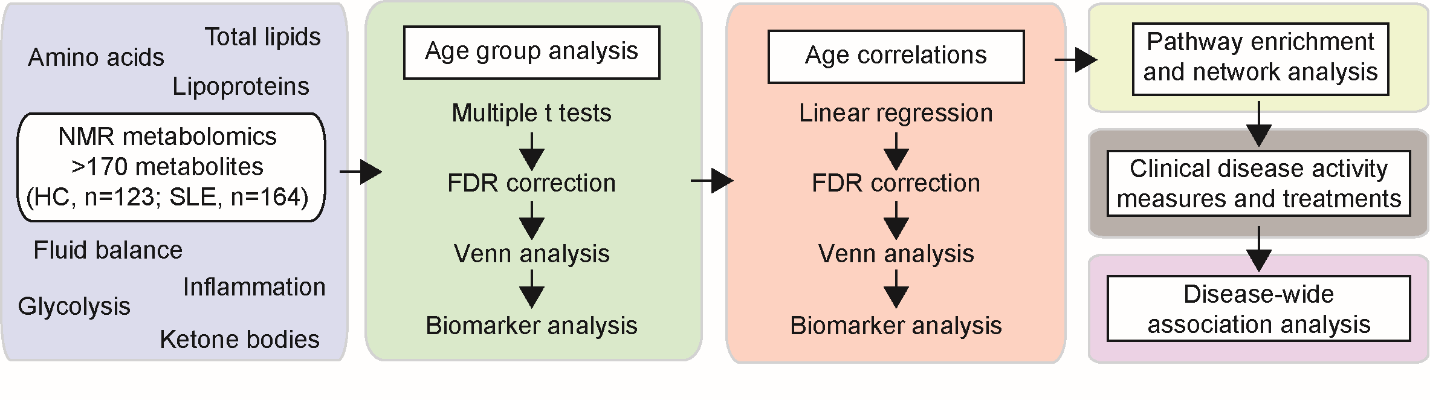
**

**Supplementary Figure S1.** Study design

**
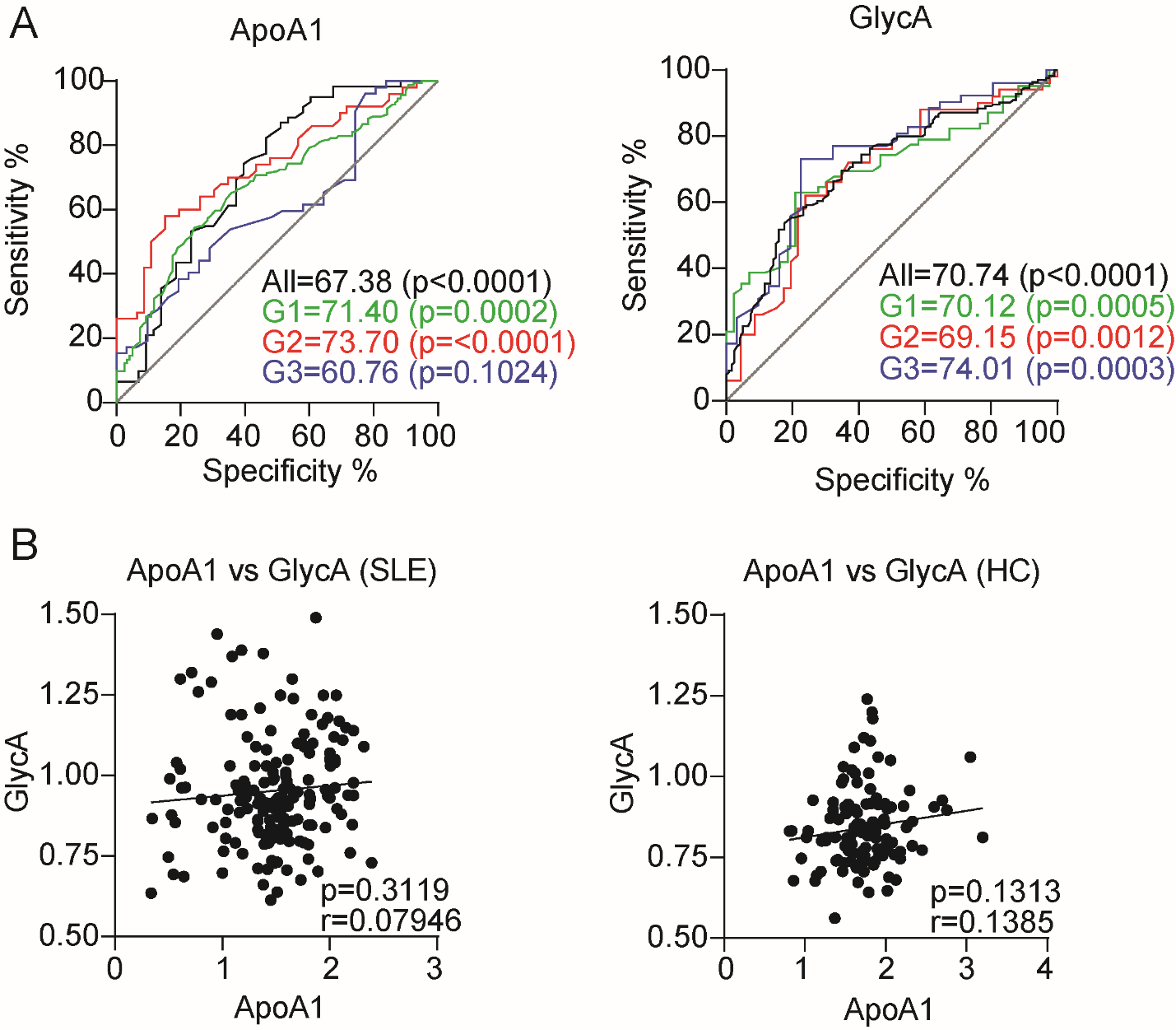
**

**Supplementary Figure S2. A)** ROC analysis comparing ApoA1 and GlycA levels in all patients, or Groups 1-3 to age-matched HCs. Area under curve (AUC) and p values are displayed. **B)** Correlation plots displaying the relationship between ApoA1 and GlycA in all SLE patients or HCs. Pearson.


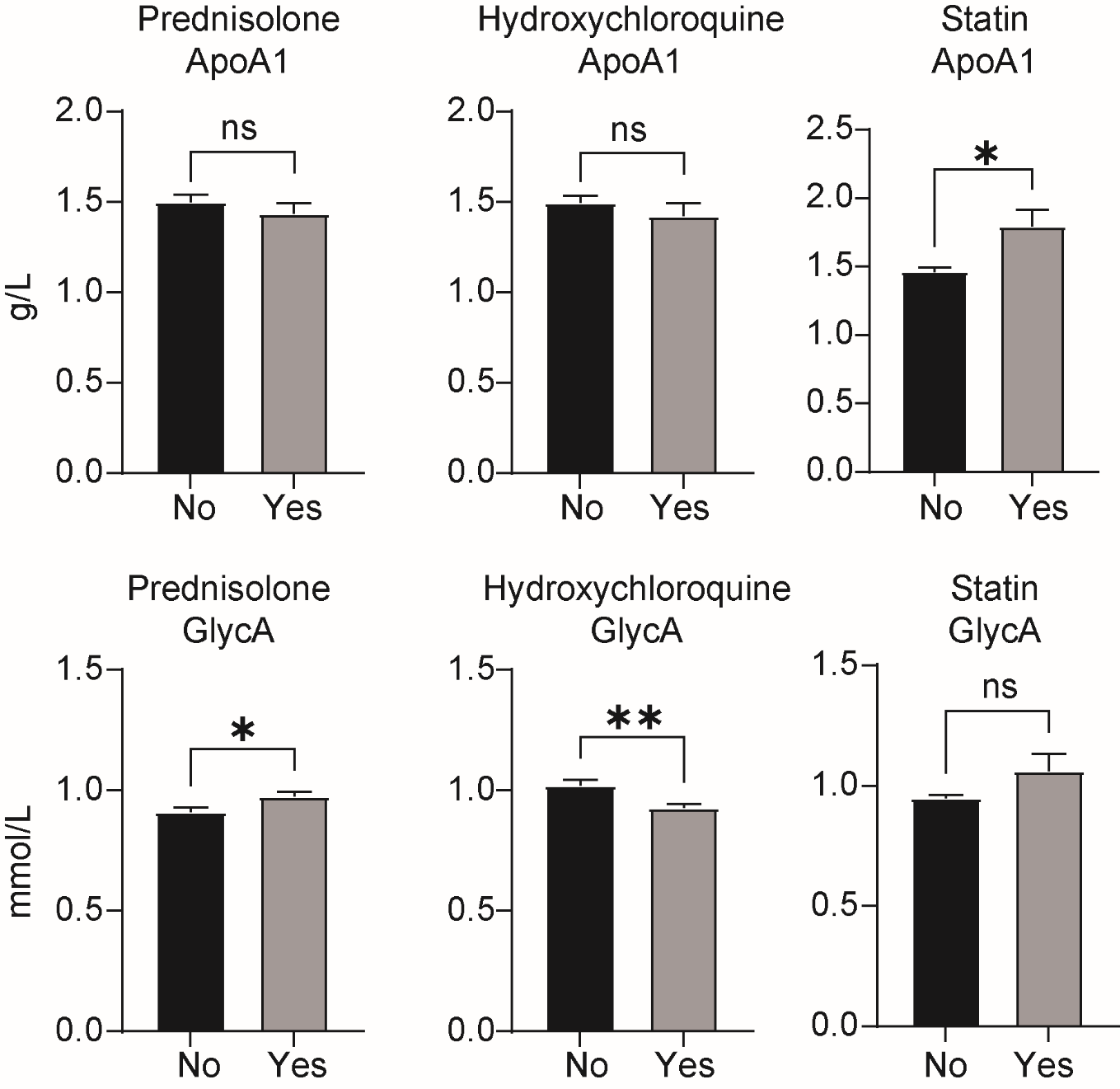


**Supplementary Figure S3.** Histograms displaying the concentration of serum ApoA1 and GlycA metabolites in SLE patients (n=164) stratified by treatment with either prednisolone (n=106) or hydroxychloroquine (n=121), or statins (n=7). *=P<0.05, **=P<0.01.


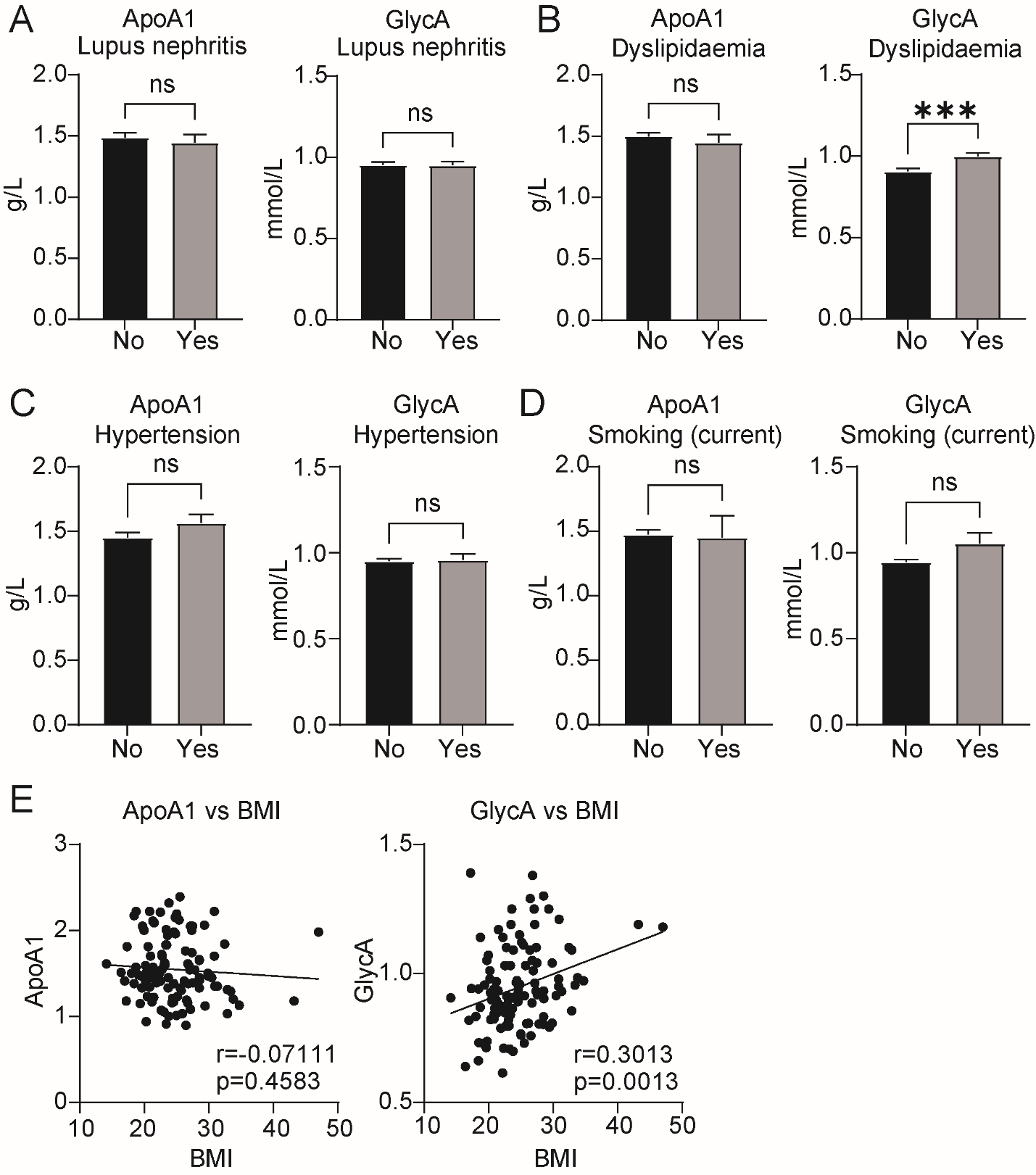


**Supplementary Figure S4. A-D)** Histograms displaying the concentration of serum ApoA1 and GlycA metabolites in SLE patients (n=164) stratified by those with **A)** lupus nephritis (n=50), **B)** dyslipidaemia (n=79), **C)** hypertension (n=30), and **D)** current smoking status (n=9). t test. ***=P<0.001. ns: not significant. **E)** Pearsons correlation comparing the levels of ApoA1 and GlycA to BMI in SLE patients.

**
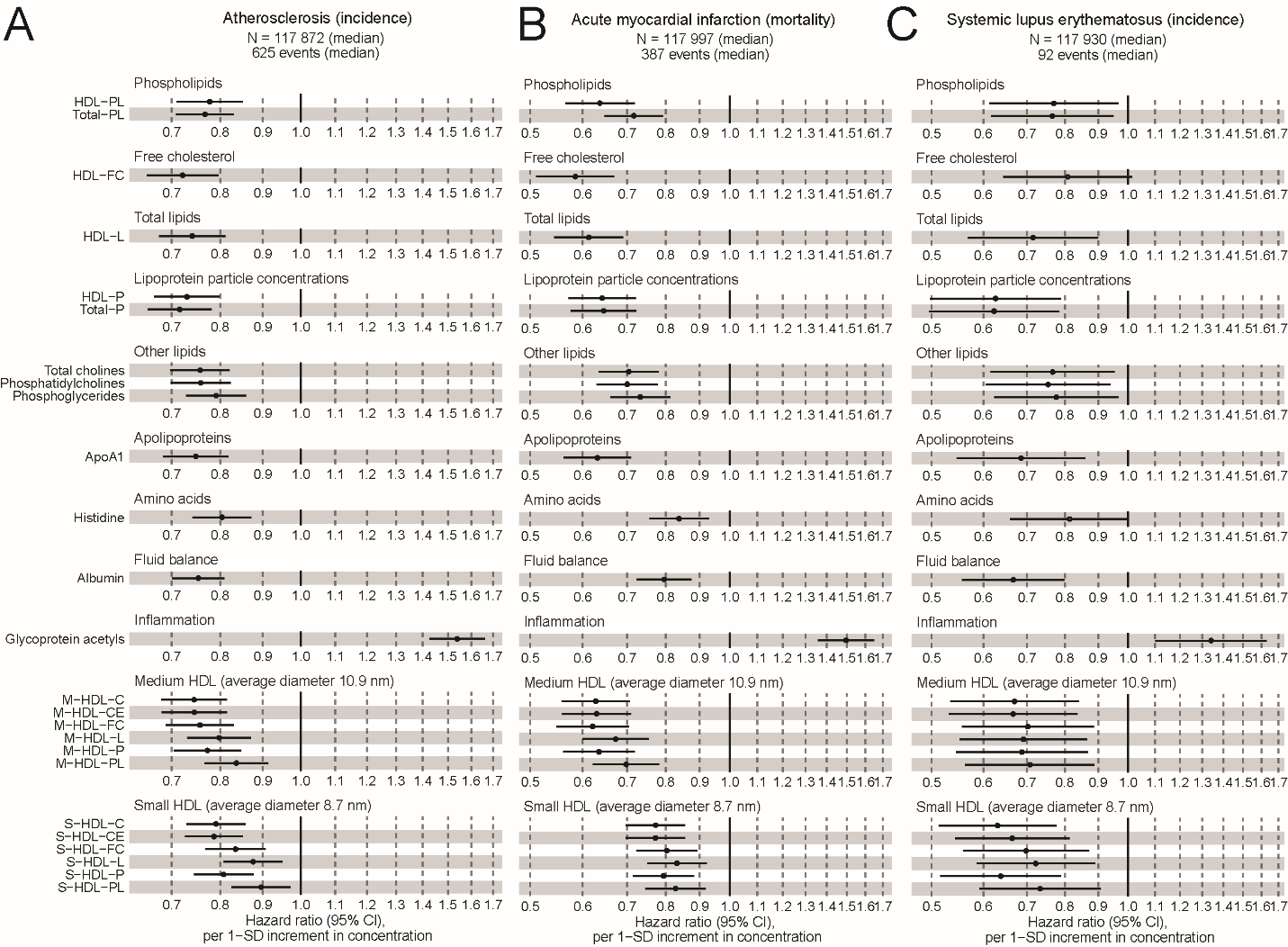
**

**Supplementary Figure S5.** Disease-wide association hazard ratio cox regression plots displaying the relationship between the 25-metaboltie signature associated with SLE in all age groups and **A)** atherosclerosis incidence, **B)** acute myocardial infarction mortality, or **C)** SLE incidence in the general population by Nightingale Atlas software, which uses open access to results from a disease-wide association scan of blood biomarkers quantified by Nightingale Health for >120,000 participants in the UK Biobank. Licensed under a Creative Commons Attribution Non-Commercial No-Derivatives 4.0 International License (CCBY-NC-ND). Hazard ratio per 1-SD increment in concentration. 95% CIs.

**
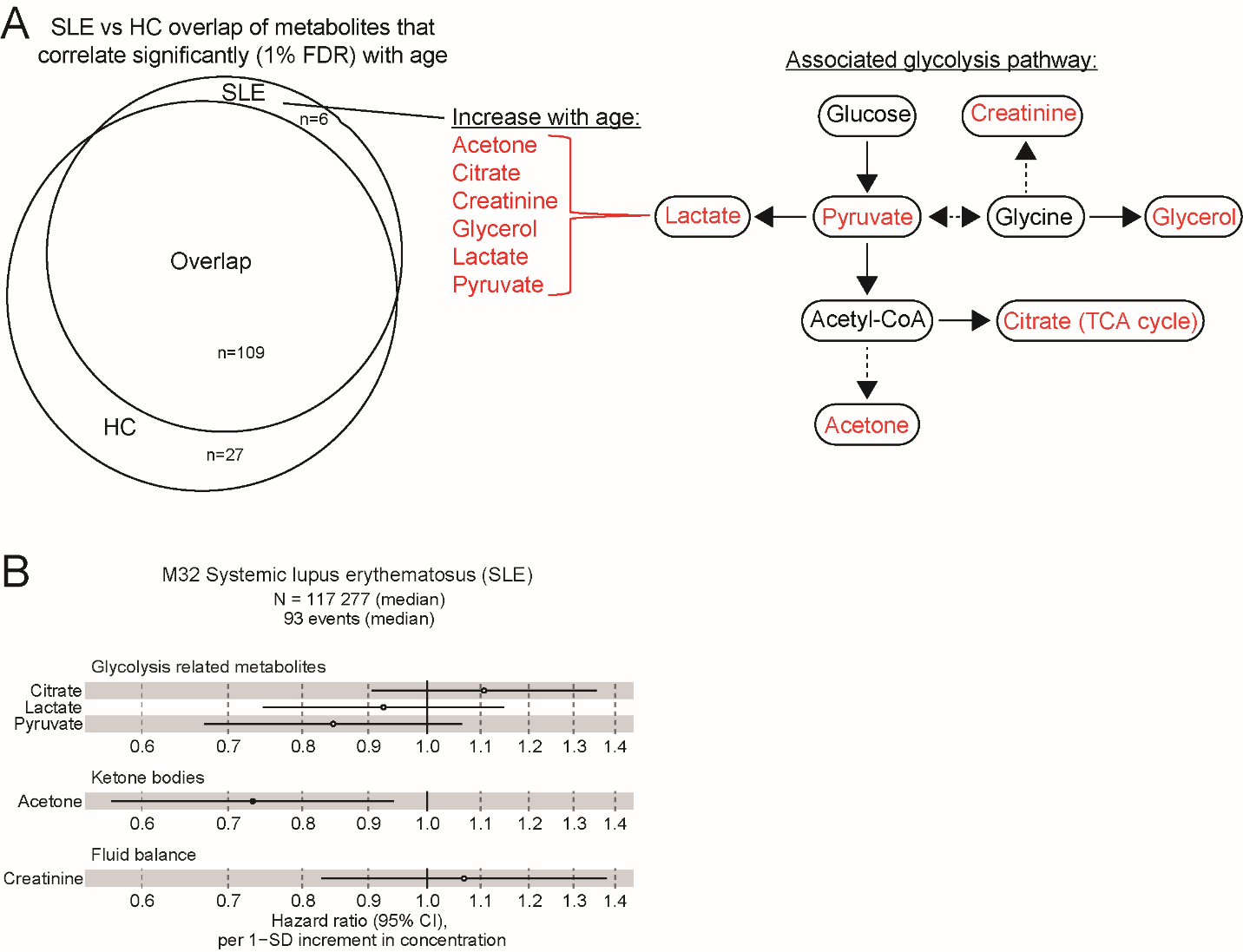
**

**Supplementary Figure S6. A-left)** Venn diagram displaying the proportional overlap of metabolites that correlate with age in SLE and/or HCs. Pearson’s correlation with false discovery rate adjustment for multiple comparisons (Benjamini, Krieger and Yekutieli approach). **A-right)** Graphical representation of the glycolysis pathway, highlighting the 6 metabolites that correlate with age in SLE only. **B)** Disease-wide association hazard ratio cox regression plots displaying the relationship between the SLE age/glycolysis-associated metabolites and SLE incidence in the general population by Nightingale Atlas software, which uses open access to results from a disease-wide association scan of blood biomarkers quantified by Nightingale Health for >120,000 participants in the UK Biobank. Licensed under a Creative Commons Attribution Non-Commercial No-Derivatives 4.0 International License (CCBY-NC-ND). Hazard ratio per 1-SD increment in concentration. 95% CIs.
